# Supplementary material for: Emulsion Polymerization Using an Amphiphilic Oligoether Ionic Liquid as a Surfactant
Source: Polymers (Basel). 2022 Aug 25;14(17):3475. doi: 10.3390/polym14173475 (PMC9459948; doi:10.3390/polym14173475)
Supplement: Supplementary file 1 [file polymers-14-03475-s001.zip › polymers-1869636-supplementary.pdf]

Supporting Information for

# EMULSION POLYMERIZATION USING IONIC LIQUID C1EG™ AND DTAB AS SURFACTANTS

Ariadna Jiménez-Victoria, René D. Peralta-Rodríguez, Enrique Saldívar-Guerra,Gladis Y. Cortez-Mazatán, Lluvia de Abril A. Soriano-Melgar, Carlos Guerrero-Sánchez

**Table S1.** Conversion and average diameter after 120 min of reaction.

| Reaction number | Repetition | Conversion*, % | Average* ± Std. Dev. | $D_p$*, nm | Average* ± Std. Dev. |
|---|---|---|---|---|---|
| 15 | a | 96.46 | 96.91 ± 0.63 | 65.46 | 66.21 ± 1.05 |
| | b | 97.35 | | 66.95 | |
| 19 | a | 95.94 | 96.42 ± 0.67 | 58.88 | 59.24 ± 0.51 |
| | b | 96.89 | | 59.60 | |
| 21 | a | 84.59 | 85.86 ± 1.76 | 63.72 | 64.26 ± 1.16 |
| | b | 87.87 | | 63.47 | |
| | c | 85.11 | | 65.60 | |
| 25 | a | 86.98 | 89.44 ± 3.47 | 54.33 | 53.71 ± 0.88 |
| | b | 91.89 | | 53.09 | |
| 27 | a | 97.82 | 97.60 ± 0.33 | 71.11 | 71.49 ± 0.85 |
| | b | 97.77 | | 72.47 | |
| | c | 97.22 | | 70.90 | |
| 28 | a | 90.52 | 90.25 ± 0.39 | 61.23 | 61.18 ± 0.07 |
| | b | 89.97 | | 61.13 | |
| 30 | a | 95.18 | 95.19 ± 0.01 | 61.20 | 60.90 ± 0.42 |
| | b | 95.20 | | 60.60 | |
| 32 | a | 92.07 | 92.11 ± 0.05 | 54.61 | 54.27 ± 0.48 |
| | b | 92.14 | | 53.93 | |

| | | | | | |
|---|---|---|---|---|---|
| 34 | a | 87.71 | 88.21 ± 0.71 | 69.70 | 69.58 ± 0.18 |
| | b | 88.71 | | 69.45 | |
| 36 | a | 97.04 | 94.90 ± 3.03 | 72.26 | 72.48 ± 0.31 |
| | b | 92.75 | | 72.70 | |

* Two significant decimal figures are used here. They are rounded to one significant figure in the main manuscript.

**Table S2.** Effect of surfactant type on conversion and particle diameter for styrene polymerizations.

| Monomers | Surfactant | Conversion [%] | PD [nm] |
|---|---|---|---|
| Styrene | **C1EG** | 89.7 ± 5.2 **a** | 66.3 ± 2.5 **a** |
| | **DTAB** | 92.0 ± 3.8 **a** | 58.0 ± 3.5 **b** |

* Different letters indicate significant differences (p≤0.05). ANOVA and *post hoc* Tukey's test.

**Table S3.** Effect of surfactant type on conversion and particle diameter for MMA polymerizations.

| Monomers | Surfactant | Conversion [%] | PD [nm] |
|---|---|---|---|
| MMA | **C1EG** | 96.1 ± 1.9 **a** | 68.8 ± 5.4 **a** |
| | **DTAB** | 92.1 ± 0.1 **b** | 54.3 ± 0.5 **b** |

* Different letters indicate significant differences (p≤0.05). ANOVA and *post hoc* Tukey's test.

**Table S4.** Effects of surfactant and initiator concentrations on conversion and particle diameter for styrene polymerizations with C1EG surfactant.

| **[S] ×CMC** | **Conversion [%]** | **PD [nm]** | **Initiator [mol×10$^4$]** | **Conversion [%]** | **PD [nm]** |
|---|---|---|---|---|---|
| **10.9** | 96.9 ± 0.6 **a** | 66.2 ± 1.1 **ab** | **3.85** | 96.9 ± 0.6 **a** | 66.2 ± 1.1 **ab** |
| **7.7** | 85.9 ± 1.8 **b** | 64.3 ± 1.2 **b** | **5.53** | 85.9 ± 1.8 **b** | 64.3 ± 1.2 **b** |
| **7.7** | 88.2 ± 0.7 **b** | 69.6 ± 0.2 **a** | **3.68** | 88.2 ± 0.7 **b** | 69.6 ± 0.2 **a** |

* Different letters indicate significant differences (p≤0.05). ANOVA and *post hoc* Tukey's test.

**Table S5.** Effects of surfactant and initiator concentrations on conversion and particle diameter for styrene polymerizations with DTAB surfactant.

| **[S] ×CMC** | **Conversion [%]** | **PD [nm]** | **Initiator [mol×10$^4$]** | **Conversion [%]** | **PD [nm]** |
|---|---|---|---|---|---|
| **3.5** | 96.4 ± 0.7 **a** | 59.2 ± 0.5 **a** | **3.93** | 96.4 ± 0.7 **a** | 59.2 ± 0.5 **a** |
| **3** | 89.4 ± 3.5 **a** | 53.7 ± 0.9 **b** | **3.14** | 89.4 ± 3.5 **a** | 53.7 ± 0.9 **b** |
| **2** | 90.3 ± 0.4 **a** | 61.2 ± 0.1 **a** | | | |

* Different letters indicate significant differences (p≤0.05). ANOVA and *post hoc* Tukey's test.

**Table S6.** Effects of surfactant and initiator concentrations on conversion and particle diameter for MMA polymerizations with C1EG surfactant.

| **[S] ×CMC** | **Conversion [%]** | **PD [nm]** | **Initiator [mol×10$^4$]** | **Conversion [%]** | **PD [nm]** |
|---|---|---|---|---|---|
| **7.7** | 94.9 ± 2.4 **a** | 72.5 ± 7.9 **a** | **3.68** | 94.9 ± 2.4 **a** | 72.5 ± 7.9 **a** |
| **10.1** | 97.6 ± 0.3 **a** | 71.5 ± 0.9 **a** | **3.80** | 97.6 ± 0.3 **a** | 71.5 ± 0.9 **a** |
| **7.7** | 95.2 ± 0.0 **a** | 60.9 ± 0.4 **b** | **5.57** | 95.2 ± 0.0 **a** | 60.9 ± 0.4 **b** |

* Different letters indicate significant differences (p≤0.05). ANOVA and *post hoc* Tukey's test.

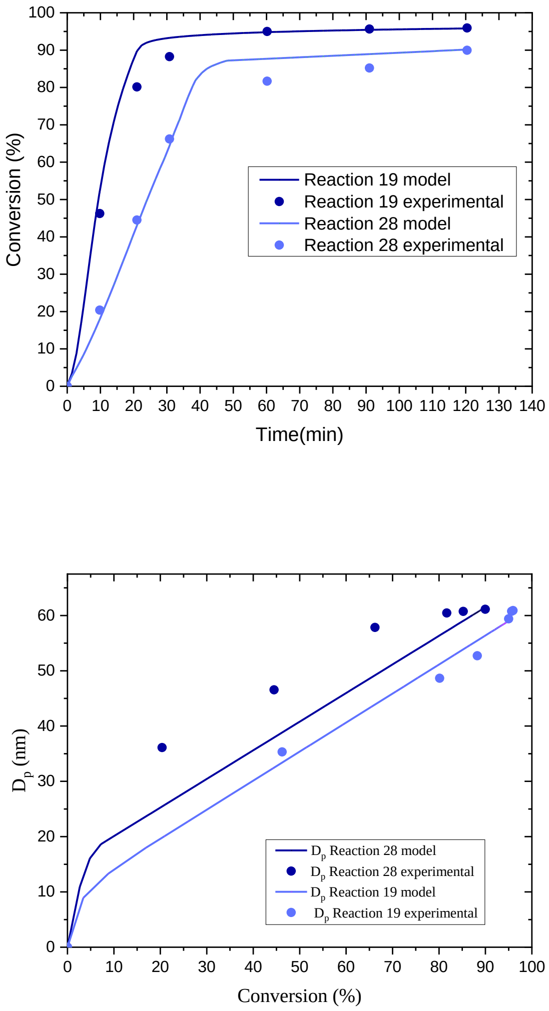


**Figure S1.** Comparison of simulation (POLYRED) and experimental data for conversion vs. time of reaction (left) and $D_p$ vs. conversion (right) for experiments E19 and E28.

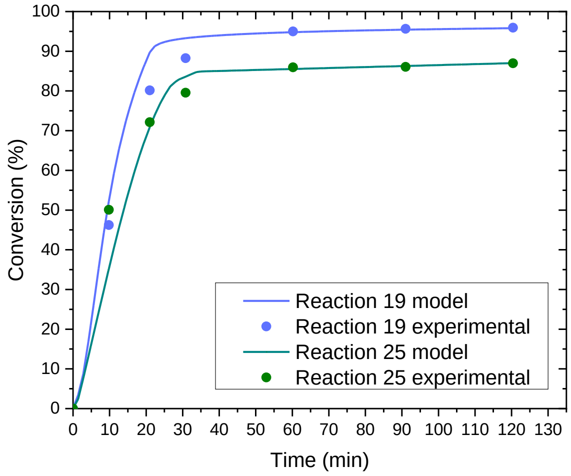


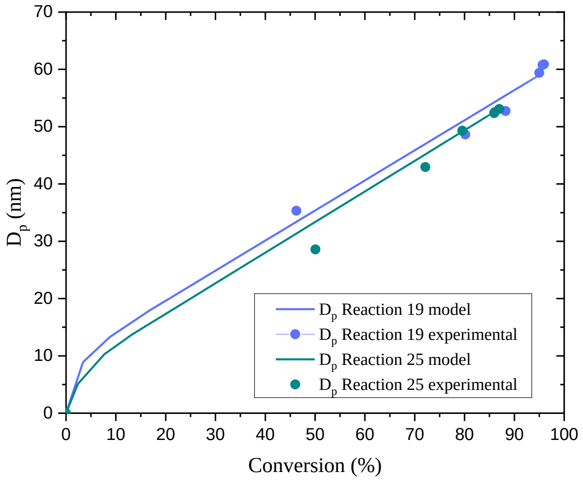


**Figure S2.** Comparison of simulation (POLYRED) and experimental data for conversion vs. time of reaction (upper) and $D_p$ vs. conversion (lower) for experiments E19 and E25.

**Table S7.** Main physical and kinetic parameters used in the POLYRED simulations (70 °C)

| **Parameter [reference]** | **Description** | **Value** |
|---|---|---|
| C1EG $r_h$ (nm) [63] | Micellar radium | 1.90 |
| DTAB $r_h$ (nm) [64] | Idem | 1.30 |
| $K_d$ ($s^{-1}$) V-50 | Initiator kinetic constant | 2.038 x $10^{+11}$ |
| $k_{p, St}$ (L $mol^{-1}$ $s^{-1}$) [65] | St propagation kinetic constant | 126 |
| $k_{p, MMA}$ (L $mol^{-1}$ $s^{-1}$) [65] | St propagation kinetic constant | 646 |
| $K_{t, MMA}$ (L $mol^{-1}$ $s^{-1}$) [66] | MMA termination kinetic constant | 1.05 x $10^{+9}$ |
| $K_{t, St}$ (L $mol^{-1}$ $s^{-1}$) [67] | St termination kinetic constant | 1.75 x $10^{+9}$ |
| $D_{P, St}$ (g $L^{-1}$) [67] | PSt density | 1054 |
| $D_{P, MMA}$ (g $L^{-1}$) [67] | PMMA density | 1170 |
| T, K | Temperature of reaction | 343.15 |
| $X_{St}$ [67] | | 4.8 |
| $X_{MMA}$ [67] | | 2.4 |

## References


[63] Sharker, K,K,; Yusa, S.; Phan, C.M. Micellar formation of cationic surfactants. *Heliyon* **2019**, *5*, e02425.

[64] Bappaditya, N.; Dey, A,; Moulik, S.P. Counter-ion effect on micellization of ionic surfactants: A comprehensive understanding with two representatives sodium dodecyl sulfate (SDS) and dodecyltrimethylammonium bromide (DTAB). *J. Surfact. Deterg.* **2013**, *16*, 785-794.

[65] Sirirat, T.; Vatanatham, T.; Hansupalak, N.; Rempel G.L.; Arayapranee, W. Kinetic study of styrene and methyl methacrylate emulsion polymerization induced by cumene hydroperoxide/tetraethylenepentamine. *J. Polym. Res.* **2014**, *22*, 2-11.

[66] Herrera-Ordóñez, J.; Olayo, R. Methyl methacrylate emulsion polymerization at low monomer concentration: Kinetic modeling of nucleation, particle size distribution, and rate of polymerization. *J. Polym. Sci. Part A Polym. Chem.* **2001**, *39*, 2547-2556.

[67] Thickett, C.; Gilbert, R.G. Emulsion polymerization: State of the art in kinetics and mechanisms. *Polymer* **2007**, *48*, 6965-6991.
